# Fluid therapy in traumatic brain injury with the resuscitation, optimization, stabilization, and evacuation (ROSE) concept approach

**DOI:** 10.62675/2965-2774.20260365

**Published:** 2026-05-08

**Authors:** Arie Zainul Fatoni, Pande Made Praskita Putra Soma, Hanggia Primadita, Ayu Yesi Agustina, Gentle Sunder Shrestha

**Affiliations:** 1 Brawijaya University Dr. Saiful Anwar General Hospital Department of Anesthesiology and Intensive Therapy Malang East Java Indonesia Department of Anesthesiology and Intensive Therapy, Dr. Saiful Anwar General Hospital, Brawijaya University - Malang, East Java, Indonesia.; 2 Tribhuvan University Teaching Hospital Department of Critical Care Medicine Maharajgunj Kathmandu Nepal Department of Critical Care Medicine, Tribhuvan University Teaching Hospital - Maharajgunj, Kathmandu, Nepal.

## INTRODUCTION

Traumatic brain injury (TBI) is a leading cause of global disability and mortality, with over 27 million new cases in 2019, underscoring the need for effective management to reduce intracranial pressure (ICP) and prevent secondary injury.^(1)^ Fluid therapy is essential and challenging, with both hypo- and hypervolemia, which could worsen cerebral perfusion and neurological outcomes.^(2)^ The ROSE concept (*R*esuscitation *O*ptimization *S*tabilization *E*vacuation) fluid management has been shown to improve the outcome of septic patients.^(3)^ This article explores applying the ROSE concept as a structured framework for fluid therapy in TBI.

## DISCUSSION

**Figure 1:**
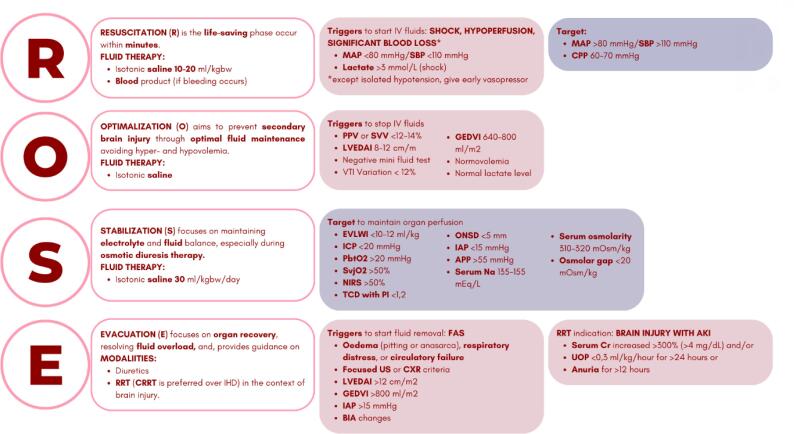
The ROSE concept adopted for the traumatic brain injury setting.

Fluid therapy in TBI aims to optimize cerebral blood flow (CBF) and oxygen delivery by restoring intravascular volume, stabilizing hemodynamics, and preserving tissue perfusion. Inappropriate fluid type or excessive volume may exacerbate cerebral edema, impair oxygen diffusion, and worsen neurological outcomes.^(3)^ A trial suggests that positive fluid balance correlates with higher intensive care unit (ICU) mortality and poorer recovery among TBI patients.^(2)^ Current review exhibits lower mortality rates, intracranial hypertension, acute kidney injury (AKI), and shorter duration of mechanical ventilation in the euvolemia group compared to positive or restrictive fluid balance in TBI patients.^(4)^ The ROSE concept could guide the physician to provide careful fluid therapy, ensuring adequate peripheral oxygen delivery and euvolemia.^(5)^ It is a conceptual model that describes four dynamic phases of fluid management based on the pathophysiology of critically ill patients (Figure 1).

MAP - mean arterial pressure; SBP - systolic blood pressure; CPP - cerebral perfusion pressure; PPV - pulse pressure variation; SVV - stroke volume variation; LVEDAI - left ventricular end-diastolic area index; VTI - velocity-time integral; GEDVI - global end-diastolic volume index; EVLWI - extravascular lung water index; ICP - intracranial pressure; PbtO_2_ - brain tissue oxygenation; SvjO_2_ - jugular venous oxygen saturation; NIRS - near-infrared spectroscopy; TCD - transcranial Doppler; ONSD - optic nerve sheath diameter; IAP - intraabdominal pressure; APP - abdominal perfusion pressure; RRT - renal replacement therapy; CRRT - continuous renal replacement therapy; FAS - fluid accumulation syndrome; US - ultrasound; CXR - chest X-ray; BIA - bioelectrical impedance analysis; UOP - urine output.

This figure is an original illustration created by the authors based on available literature.

Resuscitation (*R*) occurs within minutes of injury, when the patients enter the "ebb" phase of shock. Hypotension lowers cerebral perfusion pressure (CPP) in the injured brain and increases mortality risk 2 - 4 times, primarily due to haemorrhage. Fluid resuscitation with 15mL/kg warmed saline 0.9% can be used to increase mean arterial pressure (MAP), maintain blood osmolarity, and increase CPP while limiting cerebral edema. A recent systematic review found that 0.9% saline lowers mortality in TBI patients compared to balanced crystalloids. In severe bleeding, a 1:1:1 ratio of plasma, platelets, and red blood cells, combined with tranexamic acid, helps prevent coagulopathy.^(3,6–8)^ When blood products are unavailable, little boluses of 4mL/kg 0.9% saline may be used. However, this may be inappropriate in 12% of hypotensive TBI cases due to isolated TBI, in which hypotension results from reduced systemic vascular resistance (brain herniation, apneic phase, catecholamine depletion, or systemic inflammation) rather than hypovolemia.^(7)^ In such cases, early vasopressor treatment may be required. Colloids are discouraged. During this phase, hemodynamic targets include systolic blood pressure (SBP) ≥ 110mmHg, MAP > 80mmHg, and CPP 60 - 70mmHg.^(3,6,7)^

Optimization (*O*) extends over the first 24 hours, beginning once the patient is out of shock, but remains hemodynamically unstable. The focus is on preventing cerebral ischemia and reperfusion injury by carefully titrating maintenance fluids with small fluid boluses while avoiding fluid accumulation. Saline 0.9% remains preferred to preserve serum sodium and osmolarity to prevent brain edema. Fluid replacement or vasopressor therapy should be adjusted and guided by continuous clinical monitoring and dynamic assessment of fluid responsiveness to avoid both hypovolemia (decreased CPP and oxygen delivery) and hypervolemia (increased ICP and cerebral edema). Fluid administration should stop when indicators of adequate volume status are met, including a negative mini-fluid challenge (100 - 200mL), velocity-time integral (VTI) variation < 12%, stroke volume or pulse pressure variation < 12 - 14%, normal lactate levels, and euvolemia.^(3,9,10)^

Stabilization (*S*) spans the subsequent days, targeting homeostasis and neutral fluid balance with 0.9% saline. Monitoring should include extravascular lung water index (EVLWI) < 10mL/kg, ICP < 20mmHg, brain tissue oxygenation (PbtO_2_) > 20mmHg, jugular venous oxygen saturation (SvjO_2_) > 50%, near-infrared spectroscopy (NIRS) reading > 50%, ICP pulse morphology (P2/P1 ratio) of ≤ 1, transcranial Doppler (TCD) with a pulsatility index (PI) < 1.2, optic nerve sheath diameter (ONSD) <5 mm, intraabdominal pressure (IAP) < 15mmHg, and abdominal perfusion pressure (APP) > 55mmHg.^(3,11,12)^ In certain conditions, such as intraoperative, fluid management is maintained using the Holliday-Segar protocol. In critically ill patients, the amount of daily maintenance fluid given is 25 - 30cc/kg/day. However, in cases of polyuria, fluid replacement should be adjusted and guided by dynamic fluid responsiveness and neutral fluid balance. Hyperosmolar agents like mannitol or hypertonic saline may be indicated for intracranial hypertension to achieve and maintain serum osmolarity of 300 - 320mOsm/L, serum sodium of 135 - 155mEq/L, and osmolar gap below 20mOsm/kg. Nevertheless, their administration may complicate fluid management, thus requiring vigilant monitoring to prevent renal and electrolyte disturbances.^(3,9,13)^

Evacuation (*E*) begins immediately after resuscitation, aiming for zero fluid balance through passive or de-escalation fluid therapy. This phase is often accompanied by spontaneous diuresis as the patient recovers from critical illness (e.g., post-acute tubular necrosis). In TBI, diuresis may also result from mannitol, cerebral salt wasting syndrome (CSWS), or diabetes insipidus (DI). In these conditions, fluids must be provided to prevent hypoperfusion (4 HIT).^(3,8,14)^ Conversely, when positive fluid balance leads to multiple organ failures, known as fluid accumulation syndrome (FAS), protocolized active fluid removal with diuretics or renal replacement therapy (RRT) with net ultrafiltration should be considered. Triggers include peripheral oedema (pitting or anasarca), respiratory distress, or circulatory failure without clear cardiac or pulmonary pathology, chest X-ray abnormalities (cardiomegaly, enlarged pulmonary artery, pleural effusions, alveolar oedema, and Kerley B-lines), focused ultrasonography parameters (venous excess ultrasound score, inferior vena cava index, pleural effusion, ascites, and B-Line).^(8)^

Overzealous fluid restriction or aggressive deresuscitation could risk secondary ischemia and hemodynamic instability.^(5)^ This highlights the use of multimodal neuromonitoring as the key to successful resuscitation. Prospective multicenter trials are warranted to test the safety and effectiveness of a tailored ROSE-guided protocol in TBI.

## CONCLUSION

Fluid management in traumatic brain injury must balance cerebral perfusion and edema control. The ROSE concept is a potential framework that may help guide structured fluid management to maintain brain perfusion, minimize the risk of secondary injury, and support recovery. Further trials are necessary to establish the safety, feasibility, and clinical benefit of a tailored ROSE-guided fluid strategy in traumatic brain injury before it can be widely recommended in routine practice.

## Data Availability

Data cannot be made publicly available. This manuscript is a viewpoint article. All relevant conceptual content is fully presented within the manuscript, and no additional datasets or reusable materials are available.
